# FDA efficiency for approval process of COVID-19 therapeutics

**DOI:** 10.1186/s13027-020-00338-z

**Published:** 2020-12-01

**Authors:** Christine Cassidy, Danielle Dever, Laura Stanbery, Gerald Edelman, Lance Dworkin, John Nemunaitis

**Affiliations:** 1 Department of Medicine, University of Toledo, College and Life Sciences, Toledo, OH USA; 2 Gradalis, Inc, Carrollton, TX USA

**Keywords:** SARS-CoV-2, COVID-19, FDA, Drug development

## Abstract

Coronavirus disease 19 (COVID-19) is an infection caused by the novel Severe Acute Respiratory Syndrome Coronavirus-2 (SARS-CoV-2). The pandemic spread of SARS-CoV-2 has resulted in significant health, economic, and social ramifications. There are no U.S. Food and Drug Administration (FDA)-approved prophylactic or therapeutic treatment options for COVID-19. This puts unprecedented product development pressure on the medical science community to define treatment options. Additionally, in the United States of American (USA) further regulatory and quality assurance pressures impact the FDA. The regulatory therapeutic development process is complex as it relates to product mechanism, toxicity profile, and level of efficacy. The advert of a worldwide pandemic however, advanced efficiencies within many of the regulatory agencies worldwide in order to facilitate COVID-19 treatment option development within the USA. Clinical drug development pathways can include several established approaches: investigational new drug (IND), expanded access IND, emergency IND, treatment IND, and emergency use authorization (EUA). Remdesivir, an investigational drug, and hydroxyloroquine, an FDA-approved drug for autoimmune diseases, were the two early potential therapies. This review article examines the expedited FDA review process for remdesivir and hydroxychloroquine, and analyzes data and results from early clinical studies of both drugs.

## Background

Coronavirus disease 19 (COVID-19) began with a cluster of cases of pneumonia-like illness in Wuhan city, Hubei Province, China in December 2019 [1, 2]. The symptoms of COVID-19 vary significantly between individuals from asymptomatic to severe disease. When symptoms are detectable, they can range from mild respiratory disease to pneumonia to severe life-threatening complications characterized by acute respiratory distress syndrome (ARDS), multisystem organ failure, and death. The most common presenting symptoms include shortness of breath, fever, fatigue, myalgias, and a dry cough [3].

The first recognized cluster of COVID-19 cases in Wuhan, China presented similarly with shortness of breath, fever, dry cough, and bilateral infiltrates on lung imaging. At the time, the cases were linked to Wuhan’s Huanan Seafood Wholesale Market which sells fish and live animals such as poultry, bats, and snakes [4]. The causative virus was isolated from throat swab samples on January 7, 2020 and named Severe Acute Respiratory Syndrome Coronavirus-2 (SARS-CoV-2) by the Chinese Centre for Disease Control and Prevention (CCDC) [2, 5]. On February 11, 2020 the World Health Organization (WHO) named the disease caused by this novel coronavirus, COVID-19 [6].

COVID-19 has since spread across the globe. The first case of COVID-19 in the United States of America (USA) was diagnosed on January 20, 2020 in Washington state, and the WHO subsequently declared COVID-19 a pandemic on March 11, 2020 [7]. As of September 9, 2020 there have been 6.34 million confirmed cases in the USA and 190,065 confirmed USA deaths due to COVID-19 [8] There are currently no Food and Drug Administration (FDA)-approved prophylactic or therapeutic treatment options for COVID-19. This puts unprecedented pressure on the FDA to develop new therapies at a rapid pace.

Human coronaviruses (hCoVs) are a family of single-stranded RNA viruses that can cause respiratory, gastrointestinal, and neurological disease. They were first identified in the 1960s and are generally known as the cause of the common cold, which is relatively mild and benign [9]. However, in the past few decades, there have been three significant outbreaks of hCoVs that have caused major public health crises, which has increased the public and private sector interest in potential therapeutic developments.

SARS-CoV-2 is the third coronavirus to infect human populations in the past two decades, with the other two outbreaks being the 2002–03 Severe Acute Respiratory Distress Syndrome Coronavirus (SARS-CoV) epidemic and the 2012 Middle East Respiratory Syndrome Coronavirus (MERS-CoV) epidemic [10]. The SARS-CoV epidemic centered in Guangdong, China, presented with pneumonia-like symptoms including ARDS. It infected over 8000 people, spread across 25 countries, and had a 9.5% case fatality rate. It was declared contained by the WHO in July 2003 [11]. The MERS-CoV outbreak was traced to the Arabian Peninsula. It also presented with pneumonia-like symptoms and ARDS. It infected over 2400 people, spread across 27 countries and had a 35% case fatality rate, which is the highest of any hCoV outbreak [12]. In comparison, the COVID-19 pandemic is estimated to have a 3–4% case fatality rate, but varies by country from 1.6 to 10.7% [13].

Studies have shown that SARS-CoV-2 is very similar in structure and thus pathogenicity to SARS-CoV [14]. Both viruses rely on the spike (S) protein to bind the human angiotensin-converting enzyme 2 (hACE2) surface receptor during entry into the host cell [2, 15]. Emerging studies have shown that SARS-CoV-2 binds with a significantly higher affinity to the hACE2 receptor than SARS-CoV due to a genetic variation in the S protein. This high binding affinity supports the efficient cell entry and thus high transmissibility of SARS-CoV-2 [16]. The S protein is made up of two subunits, S1 which binds to hACE2 and S2 that facilitates fusion with the host cell membrane [15, 17]. The S protein must be cleaved by cellular proteases at the S1/S2 site in order for the S2 subunit to become active. Once the S2 subunit is active, the virus fuses with the host cell membrane [16]. These proteases include type two transmembrane serine 2 (TMPRSS2), a cell surface protease, and cathepsin B or L, lysosomal proteases. SARS-CoV-2 also possesses a furin binding site at the S1/S2 boundary which functions as another cleavage site to facilitate host cell entry, especially in cells that have downregulated TMPRSS2 or cathepsin expression [18]. Furin, an endoprotease that cleaves and proteolytically activates proproteins, is also necessary for viral egress which may have an effect on replication rate and the increased infectious rate [16, 18, 19]. The presence of the furin binding site differs from previous hCoV outbreaks, and is significant because it minimizes SARS-CoV-2’s dependence on TMPRSS2 and cathepsin B or L for host cell entry [15]. These emerging studies and hypotheses still require further investigation, but SARS-CoV-2’s affinity for hACE2 and its furin binding site may contribute to SARS-CoV-2’s increased ability to enter host cells and cause high levels of infectivity [16].

Various anti-viral therapeutic opportunities that were studied for MERS-CoV and SARS-CoV have become medical products of great renewed interest during the COVID-19 pandemic due to the genetic overlap in hCoVs [20]. Similarly, clinical trial information and data collection from the MERS-CoV and SARS-CoV epidemics have been used to guide development of therapies for SARS-CoV-2 [20].

### U.S. Food and Drug Administration therapeutic approval process

**Fig. 1:**
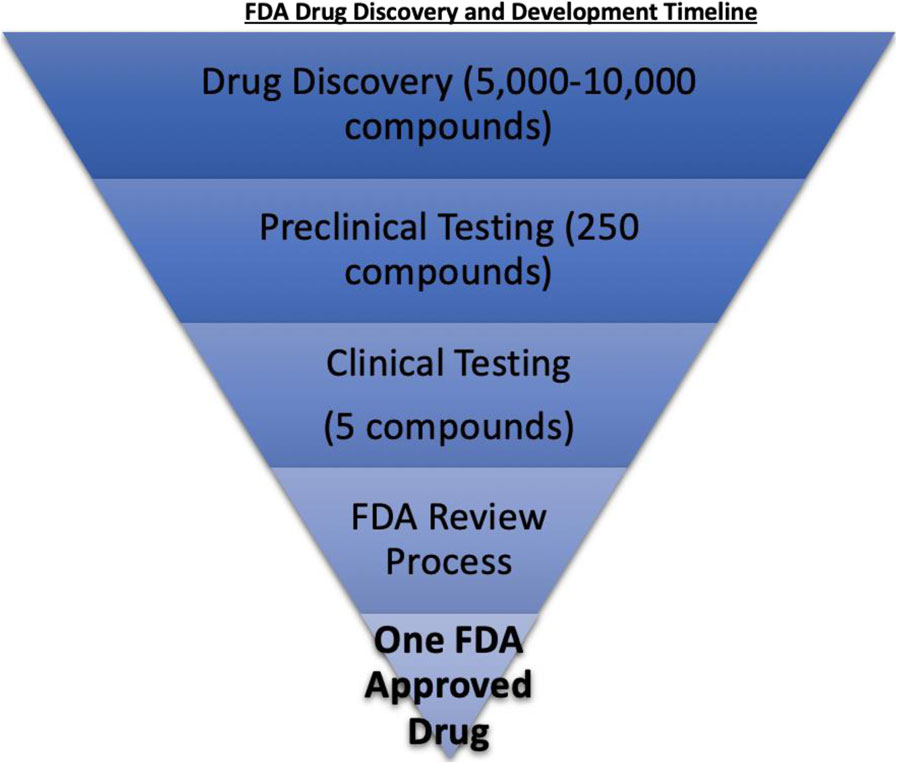
FDA Drug Discovery and Development Timeline. Thousands of candidate compounds are screened to eventually result in one FDA-approved drug. This process takes on average 10–12 years

The 1906 Pure Food and Drug Act is the foundation for the modern FDA [21]. The primary responsibility of the FDA is to ensure the safety and efficacy of medical drugs, tests, and devices delivered to consumers in the United States. A rigorous drug development process exists to uphold these standards. The traditional drug development process occurs in stages: discovery and development, preclinical research, clinical trials, FDA review, and post marketing surveillance [22]. On average, it takes 10–12 years to bring a drug from the lab bench to the consumer market in the USA [23]. Statistical studies analyzing drug development have shown only 9.6 to 13.8% of drug candidates are eventually FDA-approved (Fig. 1) [24]. The role of the FDA in drug development focuses on safety, mechanism of action, and efficacy involving clinical therapeutic use.

### Drug discovery stage

In order to submit an investigational new drug (IND) application and subsequently start clinical trials, two major phases of drug development, drug discovery and preclinical testing, must be completed [25]. While the transition to clinical investigation is clearly demarcated by the FDA issuance of an IND, the transition from drug discovery to preclinical testing is less distinct and the two phases often occur concurrently [26].

The drug discovery stage involves target selection, target validation, lead finding, and lead optimization [27, 28]. The primary goal of this phase is to identify a leading candidate compound to be further developed and eventually marketed. Investigators first identify a target gene or protein of interest that plays a significant role in a disease. Following target identification, investigators must validate that targeting the gene or protein has therapeutic benefit [27]. There are many approaches to identify and validate a target including expression profile assays (western blot), disease association genetic and expression data, analysis of molecular signaling pathways, in vivo disease models, transgenic animal models, bioactive molecules, and literature searches [27]. Once a lead candidate compound is identified, the preclinical phase can begin [26]. All data collected in this phase is used to inform and guide future studies [26].

### Preclinical stage of drug development

The preclinical drug development stage can be summarized into six preclinical milestones: 1) production of the active pharmaceutical ingredient (API); 2) pre-formulation and formulation; 3) analytical and bioanalytical methods of development and validation; 4) metabolism and pharmacokinetics; 5) toxicology; 6) good manufacturing practice (GMP) [26]. API development can occur through a variety of pathways including chemical synthesis, fermentation, biotechnology, isolation from natural sources, or a combination of methods [26]. The specific chemical and physical properties of the API must be well defined, including physical appearance, chemical impurities, stability, chirality, and enantiomers among other properties [26]. Information from this phase will be included in the IND application [25, 26]. The next stages include data collection on pharmacokinetics (PK), toxicology, and metabolism. The IND application generally requires data from two animal species (one rodent and large animal) [26]. The animal studies should mimic planned human trials as closely as possible including similar dose, duration, and frequency [26]. Toxicity studies aim to identify both the maximum tolerated dose and the no observable adverse effect level [26]. These are key endpoints to help determine the starting dose in human trials. PK data focuses primarily on determining maximum plasma concentration (C_max_), time to C_max_ (T_max_), and drug clearance [26].

**Fig. 2:**
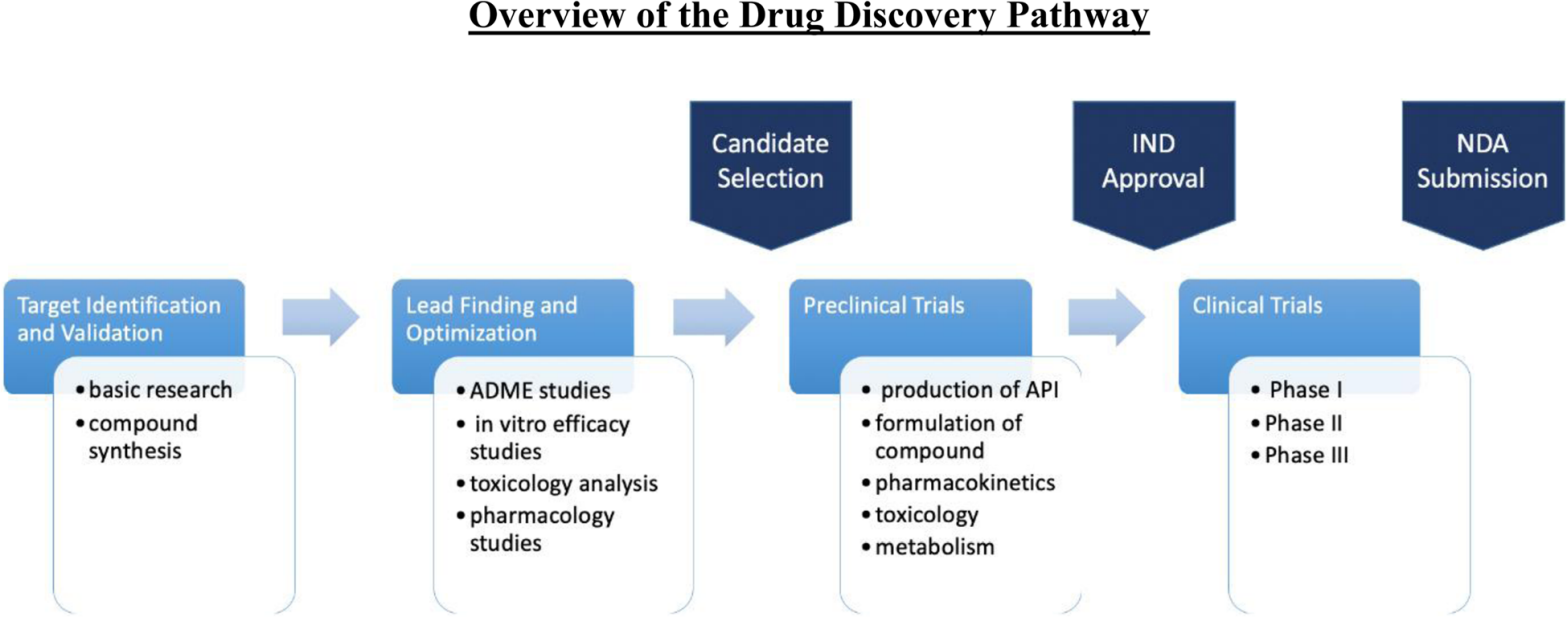
The Drug Discovery Pathway. Milestones and major checkpoints of the drug discovery process, pre-clinical, and clinical trials

Once sufficient preclinical evidence is collected to suggest clinical safety and efficacy, investigators can submit an IND application [25, 26]. The amount of data required for an IND application varies significantly depending on disease severity, available treatment options, data from compounds with a similar structure, prior human trials, and FDA-approved drugs aiming to be repurposed, among other factors [26, 27]. Furthermore, the application must provide information regarding the manufacturing process and investigator [25]. The FDA IND review team is a multidisciplinary group including a medical officer, statistician, pharmacologist, pharmacokineticist, chemist, and a microbiologist [29]. Under standard circumstances, the team has 30 days to review the IND application and make a decision to approve or deny it [30]. An overview of the process is provided in Fig. 2.

### Overview of the drug discovery pathway

#### Clinical phases of drug development

Once the IND is approved, Phase I of clinical research can begin [30]. This phase includes 20 to 100 human participants and the primary endpoint is to identify drug safety and dose. Phase II clinical trials may include several hundred participants and investigates drug efficacy and side effects. Phase III clinical trials have 300 to 3000 participants, and the aim is to compare the investigational drug to the current standard of care treatment. When both preclinical and clinical research data support drug safety and efficacy, investigators can complete a New Drug Application (NDA) through the FDA. The FDA has six to 10 months to make a decision to approve or deny the application [31]. Finally, Phase IV is considered post-market surveillance and serves to continuously monitor drug safety and efficacy [30].

**Fig. 3:**
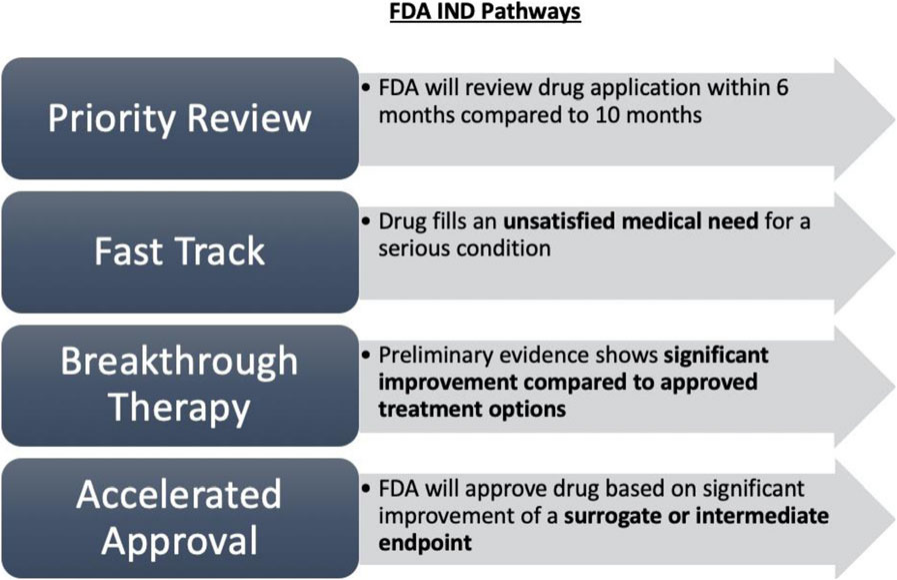
FDA Traditional IND Pathways. Highlights four strategies of the FDA to expedite traditional IND review

The traditional IND pathway described above is used to investigate an already approved drug for a new indication or an unapproved new drug [25]. Additionally, the FDA has implemented four approaches to expedite the approval of new drugs especially, drugs targeting serious diseases and diseases for which there is no treatment available. These expedited processes, within the traditional IND pathway, include Fast Track, Breakthrough Therapy, Accelerated Approval, and Priority Review (Fig. 3) [32]. These pathways are particularly relevant for COVID-19 therapeutics as many drugs are being repurposed and meet the requirements for fast track designation.

#### Types of Investigation New Drugs (INDs)

An IND can also be granted through non-traditional pathways in which patients receiving the investigational drug are not enrolled in a clinical trial [33]. To treat a single patient, a physician can request a Single Patient Expanded Access IND in non-emergent situations or an Emergency IND in life threatening situations [33, 34]. To treat more than one patient with an investigational drug outside of a clinical trial, a physician can request an Intermediate Expanded Access IND or a Treatment IND [35].

Expanded Access IND, often known as “compassionate use,” refers to the use of an investigational drug outside of a clinical trial [34]. Expanded Access INDs are considered in situations when the disease is life threatening or severely debilitating and there are no other FDA-approved, effective treatment options. Most commonly Expanded Access INDs are used for a single patient, but can also be granted for an intermediate size group (*N* > 1) of patients [35]. Approval for Expanded Access IND typically takes place within 30 days or sooner if the sponsor is contacted by the FDA and requires the following: the pharmaceutical company to supply the medication, the physician to make a formal written request to the FDA, institutional review board (IRB) approval at the site, and informed patient consent. Expanded Access IND can also be granted on an emergency basis known as an Emergency Use IND [34, 36]. An Emergency Use IND can be approved by telephone when an emergency situation does not permit time for the traditional IND application process [36]. It can also be used to provide drugs to patients that do not meet the criteria for a preexisting IND protocol or if there is no study protocol. In this case, IRB approval is not required before administering the medication.

When an investigational drug shows significant promise in the treatment of a life threatening condition, the FDA has certain pathways that allow patients access to the investigation drug before FDA approval has been granted [35]. If the investigational drug is intended to treat more than one patient, the investigator must choose between an expanded access IND and a treatment IND. Expanded access IND should be used when the investigational drug is being used for more than one patient but the drug is not yet being evaluated for marketing. A treatment IND is appropriate when a clinical trial is concluded and the data has been analyzed but has not undergone a full FDA review for approval. It is essentially an option to “bridge the gap” between a clinical trial with significant therapeutic benefit and bringing the drug to market [25, 36].

#### Emergency Use Authorization (EUA)

Emergency Use Authorization (EUA) was created under the Federal Food, Drug, and Cosmetic Act and recently amended under the Pandemic and All Hazards Preparedness Reauthorization Act of 2013, the twenty-first Century Cures Act of 2016, and Public Law 115–92 of 2017 [37]. In the circumstance that the Secretary of Health and Human Services declares an emergency, the FDA can issue an EUA for “an unapproved medical product or an unapproved use of an approved medical product” [38]. When an EUA is issued, the medical product is supplied to states from the Strategic National Stockpile (SNS) [39]. The following criteria must be met for the FDA to issue an EUA: serious or life threatening disease or condition, evidence of effectiveness, risk benefit analysis, no alternative treatments [37]. The FDA states, in a published guidance, that the decision to issue an EUA is “based on the totality of scientific evidence available” at that time [40]. In emergency situations, an EUA allows the FDA to permit use of an investigational drug that shows promise but lacks sufficient clinical trial evidence for FDA approval due to time constraints.

Historically, the FDA has issued EUAs during previous epidemics/pandemics including for H7N9 Influenza (2013), Coronavirus (2013), Ebola virus (2014), Enterovirus D68 (2015), and Zika virus (2016), and for the current COVID-19 pandemic [37, 41]. For example, the FDA worked quickly with the Mayo Clinic to make convalescent plasma from fully recovered COVID-19 patients available to hospitalized patients with severe COVID-19 through an EUA issued on August 23, 2020 [42, 43]. Remdesivir and hydroxychloroquine also received EUAs which led to follow up assessment via randomized trials [41, 44].

#### Pathways to COVID-19 therapeutics

Scientists have utilized existing data of the SARS-CoV-2 replication and cellular entry pathways to design COVID-19 therapeutics [9]. Repurposing of an already FDA-approved therapeutic is one of the fastest routes to identify safe and effective treatment options. Therapeutic repurposing has been used in the fight against COVID-19 [45]. For example, hydroxychloroquine, a drug used to treat malaria and autoimmune diseases, was investigated early on as a treatment option for COVID-19. Another approach to drug development is using investigational drugs that have proven preclinical efficacy and safety for similar diseases [9]. Remdesivir is a transitional drug that has shown promise in previous hCoV outbreaks and other RNA based viruses but is not FDA-approved to treat a disease. These therapies will be discussed in detail in the following sections.

#### Remdesivir: potential COVID-19 therapeutic

Remdesivir (Gilead Sciences, Inc. Foster City, California) is an example of a transitional therapeutic that had previously been investigated as an anti-viral but has not yet reached FDA approval. It is a nucleotide prodrug of the adenosine analog GS-441524, which functions as an RNA-dependent RNA polymerase inhibitor and has broad-spectrum antiviral activity against RNA viruses [9]. Remdesivir has most notably been investigated for SARS-CoV, MERS-CoV, and *filoviridae* (which causes Ebola virus disease) in preclinical and clinical studies [46]. Preclinical studies, such as pharmacokinetics have been completed, and clinical trials have investigated the efficacy of remdesivir in Ebola virus [47]. In addition to efficacy, trials investigating remdesivir for Ebola virus exhibited an adequate safety profile [48]. A 2017 study of remdesivir was found to have efficacy against SARS-CoV, MERS-CoV, and bat CoV strains indicating that it likely has promising broad-spectrum anti-hCoV activity [48]. In tissue culture studies, remdesivir had half-maximal effective concentrations (EC_50_s) of 0.069 uM for SARS-CoV, and 0.074uM for MERS-CoV [47]. In mouse models of SARS-CoV, prophylactic and therapeutic use of remdesivir reduced the lung viral load significantly such that the viral titers were reduced by > 2 orders of magnitude on postinfection day 4 or 5 [20]. In addition to the significantly reduced lung viral load, remdesivir used prophylactically or early in the disease course showed an improvement in respiratory function and clinical signs of disease [48]. While remdesivir has proven in vitro and in vivo efficacy against SARS-CoV, there is considerable genetic variability among coronaviruses due to its large genome size and the RNA viral replication process which is error prone [9]. Therefore, it is unclear whether results showing efficacy in SARS-CoV models is generalizable to SARS-CoV-2 without further investigation. Before the COVID-19 pandemic, remdesivir was a promising investigational drug with a well documented safety profile in human studies and known efficacy in preclinical studies of a variety of RNA viruses [20, 47, 48].

Initially, through the FDA’s Expanded Access IND protocols, physicians treating COVID-19 patients were able to apply to Gilead Sciences Inc. to offer individual patients remdesivir as a treatment [49]. An early study by Gilead published in the New England Journal of Medicine (NEJM) assessed the efficacy and tolerability of remdesivir in the treatment of severe COVID-19 in humans [50]. The study initially enrolled 61 patients, and 53 patients were included in the final safety and efficacy analysis. Patients were expected to receive a course of IV remdesivir for a duration of 10 days. Patients with confirmed SARS-CoV-2 by reverse transcriptase polymerase chain reaction (RT-PCR) assay in addition to indicators of severe disease were included. Severe disease was defined as oxygen saturation (SpO_2_) of 94% or less on room air, or a need for supplemental oxygen. Patients were excluded from the study if alanine transaminase/aspartate transaminase (ALT/AST) was above 5x the upper limit of normal or creatinine clearance below 30 ml/min. At baseline, 64% (34 of 53) of the patients were receiving invasive ventilation. The authors concluded that over 18 days, 68% (36 of 53) of the patients improved. Specifically, data showed 57% (17 of 30) of patients on mechanical ventilation were extubated, 47% (25 of 53) were discharged, and 13% (7 of 53) died.

Upon further analysis, there were potential pitfalls in the study [51]. Most prominently, there was no control group, which is understandable during a pandemic, but hinders the ability to draw conclusions about the initial efficacy of remdesivir. It is unclear from the listed methods how the 61 patients were selected, and the number of individual requests to Gilead Sciences Inc. is not listed. It would be reasonable to infer that the number of requests for a potential COVID-19 therapy was significant at the time. This additional information would be helpful in analyzing the results of the study. Eight patients were excluded from final analysis with seven of those excluded due to missing post-baseline information or a lack of post Day 1 clinical data, and one patient was excluded for an erroneous start date. Serious adverse events were reported in 23% (12 of 53) of patients such as multiple-organ dysfunction syndrome, septic shock, acute kidney injury, and hypotension. Another potential concern is that it may be difficult to differentiate between disease progression and adverse events due to the study drug. This is concerning because disease progression may be misattributed to drug side effects. The authors noted that the duration of therapy was not uniform throughout the cohort because 8% (4 of 53) discontinued treatment prematurely due to adverse side effects, which included worsening pre-existing renal failure, multiple organ failure, elevated liver enzyme studies, and maculopapular rash. The duration of therapy was also not uniform because patients could be discharged early due to clinical improvement. The authors did not report data or other analysis regarding days of treatment that the cohort received. Lastly, the viral load data was not collected from the patients to assess if there was an association between viral load level and remdesivir treatment. The study investigators had significant time and resource constraints amid an unprecedented pandemic, so despite some of the understandable potential drawbacks of the study, the results were overall promising and showed that remdesivir deserved further investigation as a COVID-19 treatment.

Following this initial NEJM study, many additional clinical trials of remdesivir began enrolling patients, and preliminary data from two studies led to the EUA for remdesivir [41]. This included a randomized double-blind, placebo controlled trial conducted by the National Institute of Health (NIH) and a Gilead Sciences Inc.-sponsored open-label trial [52, 53]. The NIH-sponsored Phase III study (Adaptive COVID-19 Treatment Trial (ACTT)) had a primary endpoint of time to recovery [52]. Recovery was defined as well enough for hospital discharge or return to normal activity, or hospitalization for solely infection-control purposes. The ACTT trial enrolled 1063 patients, and randomized 1059 patients with 538 to the remdesivir arm and 521 to the placebo arm. The preliminary data was released on April 28, 2020 in a NIH press release and subsequently published in NEJM on May 22, 2020 [52]. Preliminary data showed that there was a 31% faster time to recovery in hospitalized patients with advanced COVID-19 who received remdesivir compared to the placebo cohort. The median time to recovery in the remdesivir arm was 11 days vs. 15 days in the placebo arm (rate ratio for recovery, 1.32; 95% CI, 1.12 to 1.55; *P* < 0.001), by stratified log-rank test of the time to recovery with stratification by disease severity. While mortality was lower in the remdesivir group compared to placebo, the difference was not found to reach statistical significance (hazard ratio for death, 0.70; 95% CI, 0.47 to 1.04; 1059 patients) [52]. The authors concluded that remdesivir was able to effectively shorten the time to recovery for patients hospitalized with lower respiratory tract infection due to COVID-19.

In contrast to these conclusions, another study published in The Lancet on May 16, 2020 contradicted the NIH’s ACTT study’s promising results. The study was a randomized, double-blind, placebo-controlled, multi-center trial that analyzed remdesivir as a therapy for COVID-19 in hospitalized patients at 10 centers in Hubei, China [54]. Patients were stratified in a 2:1 ratio to remdesivir or placebo with stratification according to level of respiratory support. Two hundred thirty-seven patients were enrolled with 158 patients randomized to remdesivir and 79 patients randomized to placebo. The primary endpoint was time to clinical improvement up to day 28, and analysis was done on the intention to treat population. Clinical improvement was defined as a 2 point reduction in patients admission status on a 6 point scale for clinical status. The scale ranged from 1 to 6: 1 is discharged or having reached discharge criteria for 72 h, 2 is hospital admission but not requiring oxygen therapy, 3 is hospital admission for oxygen therapy but not requiring high flow or non-invasive ventilation, 4 is hospital admission for non-invasive ventilation or high flow oxygen therapy, 5 is hospital admission for extracorporeal membrane oxygenation (ECMO) or mechanical ventilation, and 6 is death. This scale was modified from a seven point scale used in the authors’ previous randomized control trial of lopinavir-ritonavir in COVID-19, and recommended by the WHO guidelines [55].

The authors concluded that remdesivir use was not associated with an overall difference in time to clinical improvement (hazard ratio 1.23 [95% CI 0·87–1·75], log rank *p* = .24) [54]. Although it was not statistically significant, patients with symptom duration of 10 days or less receiving remdesivir had a faster time to clinical improvement than those receiving placebo (hazard ratio 1.52 [0·95–2·43]). In addition, treatment was terminated early due to adverse events in 12% (18 of 155) of the patients receiving remdesivir compared to 5% (4 of 78) of the patients in the control group. Although these study conclusions do not support remdesivir as an effective therapeutic for COVID-19 patients, they indicate that further studies enrolling higher volumes of patients are needed to fully assess remdesivir’s role in COVID-19 treatment.

Due to the overall promising data over the first few months of trials, the FDA continues to support further research into remdesivir as a COVID-19 therapeutic. This is highlighted by the 53 FDA-approved clinical trials investigating remdesivir that were listed on clinicaltrials.gov as of September 11, 2020 [56]. Further, the EUA for remdesivir is still in place, and its indication was recently expanded while EUAs for other medical products have been revoked [44].

On August 28, 2020 the FDA announced the expansion of the existing EUA for remdesivir in a press release [57]. The EUA in May 2020 initially authorized remdesivir to treat adult and pediatric patients hospitalized with severe COVID-19 [41]. Severe COVID-19 was defined as SpO_2_ of less than or equal to 94% on room air, or requiring oxygen support such as supplemental oxygen, mechanical ventilation, and ECMO [41, 57]. In August 2020, the FDA expanded the indication for remdesivir to all pediatric and adult patients hospitalized with COVID-19, regardless of severity status [57]. The clinical trials presented here are summarized in Table 1.

**Table 1:** Clinical trials investigating Remdesivir

Study	Primary Outcome	Strengths	Weaknesses
Grein et al. [50]	- Clinical improvement observed in 36 of 53 patients (68%) hospitalized for severe COVID-19 who were given remdesivir	- Rapid organization of a clinical trial to evaluate remdesivir - Patient access to remdesivir therapy - Indicated more studies should be done to further analyze efficacy	- No control group - Small cohort - Unclear patient selection process - Difficult to differentiate adverse side effects from disease progression - Duration of therapy varied - Viral load data not collected
Beigel et al. [52], (ACTT Trial)	- 31% faster time to recovery in hospitalized patients with severe COVID-19 who received remdesivir compared to the placebo cohort (11 days vs 15 days, rate ratio for recovery, 1.32; 95% CI, 1.12 to 1.55; *P* < 0.001) - Non-statistically significant mortality benefit with remdesivir (hazard ratio for death, 0.70; 95% CI, 0.47 to 1.04; 1059 patients)	-Double-blind, randomized, and placebo-controlled -Large cohort (*N* = 1062) -Indicated further studies investigating a possible mortality benefit are warranted	- Not able to draw a definite conclusion on the mortality benefit of remdesivir
Wang et al. [54]	-The use of remdesivir in patients hospitalized with COVID-19 was not associated with an overall difference in time to clinical improvement (hazard ratio 1.23 [95% CI 0·87–1·75], log rank *p* = .24) indicating that remdesivir does not have statistically significant clinical benefit	- Double-blind, randomized, and placebo-controlled - The non-statistically significant reduction in time to clinical improvement with remdesivir in patients with symptom duration of 10 days or less indicated confirmation in larger studies is warranted	- Small cohort (*N* = 300)

Similarly, on July 3, 2020 Gilead Sciences Inc. announced that the European Commission granted conditional marketing authorization for remdesivir, which classifies remdesivir as an approved COVID-19 treatment for 1 year [58]. The European Commission based this decision upon a “rolling review of supporting data” starting in April 2020, including the NIH-funded ACTT trial [58, 59]. A conditional marketing authorization is reassessed after 1 year based upon additional studies. At that time, it can be removed from the market, extended for one more year of conditional marketing authorization or advanced to an unconditional marketing authorization [60]. As more data comes out regarding remdesivir in the coming months, it may also be approved by the FDA for use in the USA.

#### Hydroxychloroquine potential repurposed drug

Chloroquine was originally developed for the prophylaxis and treatment of malaria [61]. Since its development, it has been FDA approved for the treatment of systemic lupus erythematosus and rheumatoid arthritis. It has been investigated as an antiviral but is not currently FDA-approved for this indication. The mechanisms of action of chloroquine are proposed to be multifactorial. Based on preclinical studies, chloroquine inhibits viral replication and antigen processing through disruption of the endosomal lysosomal pathway [62]. The first step in viral replication through the endosomal/lysosomal pathway is the cleavage of viral glycoproteins by acid dependent proteases. Chloroquine and its analogues are weak bases, which easily become concentrated in acidic organelles such as endosomes and lysosomes. Therefore, chloroquine increases the pH of endosomes and lysosomes preventing both viral replication and antigen processing [61, 62]. Furthermore, chloroquine has been shown preclinically to have numerous anti-inflammatory effects including inhibition of antigen presentation and inhibition of cytokine production and release [61]. Another proposed mechanism of chloroquine is interference with glycosylation of hACE2, which decreases viral entry of hCoV [63]. Chloroquine was shown to prevent viral replication in vitro when given both before and after cells were infected with SARS-CoV [64]. Subsequently in animal trials, chloroquine was also shown to increase survival rates in newborn mice infected with human coronaviruses [65].

Preexisting in vitro evidence of chloroquine against viruses, specifically SARS-CoV, made chloroquine and hydroxychloroquine, its analog, a plausible option when SARS-CoV-2 emerged in China in December 2019 [64]. Evidence rapidly accumulated supporting the efficacy of chloroquine and hydroxychloroquine in vitro against SARS-CoV-2 specifically [66]. Yao et al. showed hydroxychloroquine, a chloroquine analog with a more favorable safety profile, was more potent against SARS-CoV-2 than chloroquine in vitro. Yao et al. was also the first to suggest a dosing regimen for hydroxychloroquine in the treatment of COVID-19 [67]. This drug was an attractive choice for the treatment of COVID-19 because it showed in vitro evidence against SARS-CoV-2 specifically and was already FDA-approved for other indications with a well-documented safety profile and post market surveillance.

The first clinical results of chloroquine efficacy in the treatment of COVID-19 were reported in a news briefing by the State Council of China in February of 2020 (Table 2) [45]. Based on results from multiple clinical trials across China, scientists reported superior outcomes in over 100 patients that received chloroquine. The outcomes reported included pneumonia exacerbation, lung imaging, and disease course. Importantly, no adverse events were reported. To communicate superior outcomes quickly, the results were reported as part of a news briefing rather than through the traditional data publication and peer review process. While this limited the ability to analyze the results, the information clearly indicated that more trials were needed to investigate chloroquine in the treatment of COVID-19.

**Table 2:** Clinic trials investigating the efficacy of hydroxychloroquine for COVID-19

Study	Primary Outcome	Strengths	Weaknesses
Gao et al. [45]	- Compared to control treatment, more than 100 patients treated with chloroquine had superior inhibition of pneumonia exacerbation, negative seroconversion, and a shortened disease course	- Rapid action to organize a clinical trial for a novel disease based on in vitro data - Reported both virologic and clinical outcomes - Indicated more trials were needed	- Clinical trial results reported as part of a news briefing by the State Council of China rather than in a peer reviewed journal
Gautret et al. [68]	- Statistically significant more patients treated with hydroxychloroquine (14/20 = 70%) had negative nasopharyngeal PCR for SARS-CoV-2 by day 6 post inclusion compared to controls (2/16 = 12.5%) (*p* = 0.001)	- First clinical evidence on the effect of hydroxychloroquine on the nasopharyngeal clearance of SARS-CoV-2 with reported mean duration of viral shedding to be 20 days - Indicated more trials were needed to investigate the clinical effects of hydroxychloroquine	- Non-randomized - Wide range of severity of illness - Combination therapy with azithromycin in six patients - Small sample size (*N* = 36) - 6 of 26 patients in HCQ treatment group were lost to follow up including ICU admission (3), death (1), leaving hospital (1), and cessation of treatment due to nausea (1)
Chen et al. [69]	- There was no statistically significant difference between the hydroxychloroquine group (86.7%) and control group (93.3%) in day 7 negative nucleic acid throat swab for SARS-CoV-2 in hospitalized patients with COVID-19 (*p* > 0.05)	- Randomization of participants - Reported clinical endpoints in addition to virologic clearance	- Small sample size (N = 30)
Tang et al. [70]	- There was no significant difference in negative conversion of SARS-CoV-2 by 28 days between hydroxychloroquine and the standard of care group in patients with mild to moderate COVID-19 with higher adverse events in the hydroxychloroquine group	- Randomization - Increased sample size (*N* = 150) - Included patients with mainly mild to moderate disease (148/150) - Intention to treat statistical analysis	
Horby et al., [71] (RECOVERY Trial)	- There was no statistically significant difference in mortality between the hydroxychloroquine arm and the standard of care arm - Enrollment suspended from hydroxychloroquine arm on 6/5/20	- Randomized, multiarmed clinical trial - Large sample size with 1542 patients in the hydroxychloroquine arm - Primary endpoint of 28-day mortality	
Pan et al., [72] (SOLIDARITY Trial)	- There was no statistically significant effect on hospitalized patients with COVID-19 including overall mortality - Hydroxychloroquine arm dropped from trial on 6/17/20	- Randomized, international clinical trial - Large sample size with 954 patients in the hydroxychloroquine arm - Investigated hospitalized population	

Shortly after this press briefing, the results of an open label, non-randomized clinical trial in France were published [68]. A total of 20 patients with confirmed COVID-19 were treated with 600 mg of hydroxychloroquine per day. Six of the 20 patients in the treatment group also received azithromycin based on clinical judgement. Sixteen patients were enrolled in the control group. The severity of illness in trial participants varied significantly, ranging from asymptomatic to severe respiratory tract infection requiring intensive care unit (ICU) admission. The primary endpoint was virological clearance by day 6 of treatment. This endpoint was met in 70% (14 of 20) of patients in the treatment group compared to 12.5% (2 of 16) in the control group (*p* = 0.001). However, there were several limitations to this study including a small sample size. Furthermore, six patients were not included in the data analysis due to loss to follow up. Lost to follow up was defined as ICU admission within the same hospital in three patients, death in one patient, withdrawal of treatment in one patient, and leaving against medical advice in one patient. The results of this trial were promising in this unprecedented pandemic and encouraged the scientific community that more trials were needed to investigate hydroxychloroquine.

A randomized clinical trial in Japan investigating the effect of hydroxychloroquine on COVID-19 used a similar endpoint to the trial in France, virological clearance, indicated by negative SARS-CoV-2 virus detection from pharyngeal swabs on day seven of treatment with hydroxychloroquine [69]. In this clinical trial, 30 patients were randomized 1:1 to receive either 400 mg hydroxychloroquine plus conventional treatment or conventional treatment alone. Virologic clearance on day 7 was reported in 86.7% (13 of 15) of patients in the hydroxychloroquine treatment group compared to the 93.3% (14 of 15) of patients in the control group (*p* > 0.05), therefore the results were not statistically significant. Additionally, there were no significant differences between groups in median duration from hospitalization to negative conversion or median time for temperature normalization. Although these studies had similar designs and endpoints, the results were conflicting. Furthermore, the endpoint of virological clearance did not always correlate with patient clinical status and ultimate recovery, informing the medical community that more trials with clinical endpoints were necessary.

As confirmed COVID-19 cases rose in the USA, available clinical data was limited, making it difficult for physicians to understand the risks and benefits of hydroxychloroquine for their COVID-19 patients [68, 69]. On March 28, 2020 the FDA issued an EUA for the use of hydroxychloroquine and chloroquine for the treatment of COVID-19 in hospitalized patients [44]. As described previously, the EUA allowed physicians to use hydroxychloroquine as a COVID-19 therapeutic, which is an unapproved use of an approved medical product when participation in a clinical trial is not feasible [37].

Clinicians quickly called for randomized clinical trials to conclusively determine the efficacy of hydroxychloroquine in the treatment of COVID-19. As of June 2020 there were 230 clinical trials registered on clinicaltrials.gov investigating hydroxychloroquine [73]. An open label, randomized controlled trial of 150 hospitalized patients with COVID-19 published in May 2020 investigated negative conversion of SARS-CoV-2 by day 28 of treatment in patients with mild to moderate disease [70]. The treatment group received a loading dose of 1200 mg of hydroxychloroquine per day for 3 days and a maintenance dose of 800 mg per day for two to 3 weeks. Mild disease was defined as mild symptoms without pneumonia on imaging. Moderate disease was defined as fever, cough with sputum production, pneumonia on imaging but no signs of severe pneumonia with SaO_2_ less than 94% on room air or a partial pressure of oxygen (PaO_2_) to fraction of inspired oxygen (FiO_2_) ratio of less than or equal to 300. There was no significant difference between groups in the probability of negative conversion by day 28 of treatment. Furthermore, there were more adverse events in the hydroxychloroquine group (30%, 21 of 70) compared to the control group (9%, 7 of 80). Serious adverse events occurred in 3% (2 of 70) of patients in the hydroxychloroquine group and 0% of patients in the standard of care group. Serious adverse events were listed as disease progression and upper respiratory tract infection. Non-serious adverse events included diarrhea, thirst, and transient blurred vision. Diarrhea was the most common adverse event occurring in 10% (7 of 70) of patients in the hydroxychloroquine group and no patients in the standard of care group. Hydroxychloroquine was discontinued in one patient due to blurred vision and one patient due to reported thirst, both of which resolved within 2 days of discontinuing the drug. Although arrhythmias are a well-documented side effect of hydroxychloroquine, no patients in this trial had any observable serious events of cardiac arrhythmias [70, 74].

The RECOVERY Trial, is a randomized multiarmed clinical trial in the United Kingdom investigating multiple drugs for the treatment of COVID-19 [71]. In this trial 1542 patients were randomized to receive hydroxychloroquine and the primary endpoint was 28-day mortality. Preliminary data showed no significant difference in mortality between the hydroxychloroquine group (25.7%) and the standard of care group (23.5%) (hazard ratio 1.11 [95% confidence interval 0.98–1.26]; *p* = 0.10). With this preliminary data, the decision was made to suspend enrollment in the hydroxychloroquine arm of the trial on June 5, 2020 [75]. On June 15, 2020 the FDA revoked the EUA for hydroxychloroquine as the required legal criteria were no longer met [76]. Due to accumulating data on the serious cardiac adverse events, the FDA determined the potential benefit no longer outweighed the risks of the medication [77]. The WHO followed on June 17, 2020 discontinuing the hydroxychloroquine arm of the Solidarity Trial [72]. The Solidarity Trial is an international clinical trial comparing treatment options for hospitalized patients with COVID-19. The hydroxychloroquine only arm was dropped from the trial in June 2020. The current treatment options in the trial include remdesivir only and lopinavir-ritonavir with interferon beta 1a [72].

Hydroxychloroquine in the treatment of COVID-19 is an example of a repurposed FDA-approved therapeutic. Hydroxychloroquine was the first drug of the pandemic that was issued an EUA by the FDA [44]. While the in vitro and preclinical evidence was promising, demonstration of clinical benefit has been limited. A major advantage of this pathway is knowledge of adverse effects and safety profile. While clinicians did not know if hydroxychloroquine would be effective in the treatment of COVID-19, they knew which patients should be excluded from receiving the medication and what adverse events to expect. Although the EUA for hydroxychloroquine was eventually revoked, the EUA allowed COVID-19 patients access to a potentially promising treatment in a time when no FDA-approved treatment options existed. In addition, the EUA made hydroxychloroquine readily accessible to patients which in turn allowed for significant data collection, research trials, and data analysis on hydroxychloroquine in humans with COVID-19. This only helped physicians, scientists, and the FDA to make early conclusions about effective vs non-effective treatments for COVID-19.

#### Regulatory efficiency modifications

Due to the time constraints and pressure to find effective COVID-19 therapies quickly, the majority of the studies described in this review were underpowered and often did not have a control group. This makes the statistical analysis difficult to interpret and apply to clinical practice. Despite these shortcomings, the rapid expansion of trials were overall able to help scientists draw important early conclusions, such as remdesivir warranted further investigation and hydroxychloroquine was likely not as effective as preclinical testing suggested [50, 64, 65, 71, 72]. The most impressive part of these studies was the ability of the FDA to get them running quickly and to collect data points to guide future studies rapidly as well.

As seen in the trials described throughout this review, the emergent threat to humanity posed by the COVID-19 pandemic placed unprecedented pressure on the FDA to work closely with trial investigators, researchers, and drug companies in the private sector to bring effective therapeutics to the bedside as quickly and safely as possible. The FDA acted rapidly to increase access to treatment for patients in the USA, most significantly through the creation of the Coronavirus Treatment Acceleration Program (CTAP) and the formation of the Accelerating COVID-19 Therapeutic Interventions and Vaccines (ACTIV) partnership between the private and public sectors [78, 79]. The purpose of the CTAP was to provide very rapid review of potential treatments and make them available to patients as fast as possible, while maintaining patient safety [78]. The FDA aimed to respond to protocol reviews within 24 h of submission, finalize the review process of single patient expanded access IND requests within 3 h, and work closely with drug developers to approve studies. As of September 2020, the FDA has approved 310 trials of therapeutic agents and there are 590 development programs for therapies in the planning and design stages that are working with the FDA. This rapid acceleration of clinical trial development proves the power of the FDA when it focuses its resources and manpower on a specific problem. This diverges from the sometimes arduous process that many scientists are familiar with when working to investigate a new medical product. In addition, the ACTIV partnership includes the NIH, FDA, and Centers for Disease Control from the public sector, and major pharmaceutical companies like Bristol Myers Squibb, Eli Lily and Company, Merck & Co., Pfizer, and Roche among others [79]. The overarching goal of this partnership is to create infrastructure, combine expertise, and provide funding to identify the most promising compounds to prioritize their testing and development. The creation of the CTAP and ACTIV by the FDA has allowed for expedited COVID-19 therapeutic development and could be similarly applied to other diseases with limited treatment options and high mortality. As an example, cancer is a leading cause of death in the USA and approximately 606,880 people died from the disease in 2019, which is more than 3x the number of current COVID-19 deaths [80] and about 38% of people will be diagnosed with cancer at some point in their life, compared to 1.3% of the USA population diagnosed with COVID-19 [81]. Consideration of similar aggressive approaches as a focus in cancer or other severely debilitating disease product development may be an attractive future opportunity.

## Data Availability

N/A

## Abbreviations

COVID-19: Coronavirus disease 19
ARDS: Acute respiratory distress
CCDC: Chinese Center for Disease Control and Prevention
WHO: World Health Organization
FDA: Food and Drug Administration
hCoVs: Human coronaviruses
SARS-CoV: Severe Acute Respiratory Distress Syndrome Coronavirus
MERS-CoV: Middle East Respiratory Syndrome Coronavirus
TMPRSS2: Type tow transmembrane serine 2
ADME: Absorption, distribution, metabolism and excretion
API: Active pharmaceutical ingredient
GMP: Good manufacturing practice
DP: Drug product
PK: Pharmacokinetics
NHP: Nonhuman primate
NDA: New drug application
IND: Investigational new drug
EUA: Emergency use authorization
RT-PCR: Reverse transcriptase polymerase reaction
SpO_2_: Oxygen saturation
ALT: Alanine transaminase
AST: Aspartate transaminase
ACTT: Adaptive COVID-19 Treatment Trial
ICU: Intensive care unit
CTAP: Coronavirus Treatment Acceleration Program
ACTIV: COVID-19 Therapeutic Interventions and Vaccines
